# Supplementary material for: Upregulated Guanine Deaminase Is Involved in Hyperpigmentation of Seborrheic Keratosis via Uric Acid Release
Source: Int J Mol Sci. 2021 Nov 19;22(22):12501. doi: 10.3390/ijms222212501 (PMC8625227; doi:10.3390/ijms222212501)
Supplement: Supplementary file 1 [file ijms-22-12501-s001.zip › ijms-1359835-supplementary.pdf]

International Journal of *Molecular Sciences*

MDPI

Article

Upregulated Guanine Deaminase Is Involved in Hyperpigmentation of Seborrheic Keratosis via Uric Acid Release

Kyung Ah Cheong [1], In Sup Kil [2], Hyuk Wan Ko [3] and Ai-Young Lee [1,*]

[1] Department of Dermatology, Dongguk University Ilsan Hospital, 814 Siksa-dong, Ilsandong-gu, Goyang-si, 410-773, Gyeonggi-do, Korea; bionase@hanmail.net
[2] Basic Research & Innovation Division, Amorepacific Corporation R&D Center, Yongin-si, 446-729 Gyeonggi-do, Korea; iskil@amorepacific.com
[3] Department of Biochemistry, College of Life Science and Biotechnology, Yonsei University, Seoul 03722, Korea; kohw@yonsei.ac.kr
* Correspondence: lay5604@naver.com; Tel.: +82-319-617-250

Supplementary file

ELISA assay

Samples for ELISA assay were prepared from supernatant of keratinocytes with or without transfected GDA. Concentrations of b-FGF and SCF were determined using ELISA kits (R&D System, Minneapolis, MN, USA).

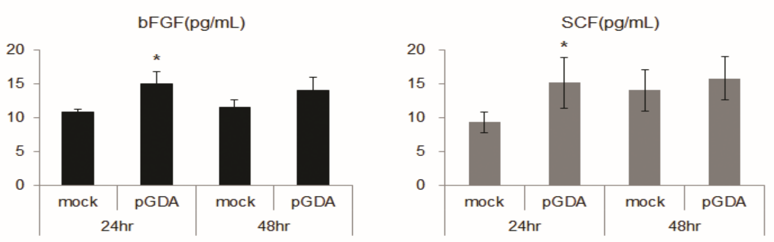

Figure S1. Growth factor generation by *GDA*-overexpressing keratinocytes.

ELISA was performed to determine concentrations of bFGF and SCF proteins in culture supernatants from viable control gene and *GDA*-overexpressing keratinocytes. Data represent means ± SD of four independent experiments. * $p < 0.05$ *vs.GDA*-overexpressing keratinocytes.
